# Central Apolipoprotein A-IV Stimulates Thermogenesis in Brown Adipose Tissue

**DOI:** 10.3390/ijms22031221

**Published:** 2021-01-27

**Authors:** Sydney Pence, Zachary LaRussa, Zhijun Shen, Min Liu, Karen T. Coschigano, Haifei Shi, Chunmin C. Lo

**Affiliations:** 1 Diabetes Institute and Honor Tutorial College, Department of Biomedical Sciences, Heritage College of Osteopathic Medicine, Ohio University, Athens, OH 45701, USA; sp883713@ohio.edu (S.P.); zl928415@ohio.edu (Z.L.); shenzusa@hotmail.com (Z.S.); coschigk@ohio.edu (K.T.C.); 2 Department of Pathology and Laboratory Medicine, Metabolic Diseases Institute, University of Cincinnati, Cincinnati, OH 45221, USA; lium@ucmail.uc.edu; 3 Department of Biology, Miami University, Oxford, OH 45056, USA; shih@miamioh.edu

**Keywords:** third ventricle, unilateral denervation, thermogenesis, brown adipose tissue

## Abstract

Stimulation of thermogenesis in brown adipose tissue (BAT) could have far-reaching health benefits in combatting obesity and obesity-related complications. Apolipoprotein A-IV (ApoA-IV), produced by the gut and the brain in the presence of dietary lipids, is a well-known short-term satiating protein. While our previous studies have demonstrated reduced diet-induced thermogenesis in ApoA-IV-deficient mice, it is unclear whether this reduction is due to a loss of peripheral or central effects of ApoA-IV. We hypothesized that central administration of ApoA-IV stimulates BAT thermogenesis and that sympathetic and sensory innervation is necessary for this action. To test this hypothesis, mice with unilateral denervation of interscapular BAT received central injections of recombinant ApoA-IV protein or artificial cerebrospinal fluid (CSF). The effects of central ApoA-IV on BAT temperature and thermogenesis in mice with unilateral denervation of the intrascapular BAT were monitored using transponder probe implantation, qPCR, and immunoblots. Relative to CSF, central administration of ApoA-IV significantly increased temperature and UCP expression in BAT. However, all of these effects were significantly attenuated or prevented in mice with unilateral denervation. Together, these results clearly demonstrate that ApoA-IV regulates BAT thermogenesis centrally, and this effect is mediated through sympathetic and sensory nerves.

## 1. Introduction

Obesity has become a global epidemic and affects more than 30% of the world’s population [1]. In parallel, the incidence of type 2 diabetes, osteoarthritis, chronic kidney disease, many types of cancers, and coronary heart disease has increased [2]. Stimulation of brown adipose tissue (BAT) has the potential to reduce obesity, insulin resistance, and obesity-related cardiovascular diseases in humans [3]. Although whether BAT is a realistic pharmaceutical target for treating obesity in humans awaits to be further confirmed [4,5], identifying a potent stimulant of BAT thermogenesis may provide a promising avenue for development of new strategies to combat obesity and obesity-related diseases.

Dietary lipids stimulate BAT thermogenesis through activation of gut–brain–BAT neurocircuits in lean animals and humans to counteract the energy surplus [6,7,8]. Dietary lipids increase pro-opiomelanocortin (POMC) expression and activate the melanocortin system in the hypothalamus [9,10], leading to the inhibition of food intake [11] and the induction of norepinephrine (NE) from sympathetic nerves in BAT [12,13,14]. NE binds to β3-adrenergic receptors and enhances intracellular lipolysis in BAT [15]. The free fatty acids are then taken into the mitochondrial matrix and oxidized to promote diet-induced BAT thermogenesis with the assistance of UCP1, leading to a decrease in body weight gain [14]. In contrast, chronic consumption of a high-fat diet (HFD) for more than three weeks does not promote diet-induced BAT thermogenesis due to decreased sympathetic activity [16,17,18,19], contributing to the development of obesity.

Apolipoprotein A-IV (ApoA-IV) is highly present in the neurons of the hypothalamus [20,21]. Short-term consumption of dietary lipids increases hypothalamic ApoA-IV levels in animals [22,23]. However, chronic consumption of HFD downregulates hypothalamic expression of ApoA-IV [16,17,18,19]. In the small intestine, the synthesis and secretion of ApoA-IV is associated with the transport of long-chain fatty acids and lipid intake [24,25,26]. Once in the circulation, ApoA-IV hydrolyzes circulating triglyceride (TG) through activation of lipoprotein lipase [27,28]. In addition, ApoA-IV activates lecithin–cholesterol acyltransferase, increasing cholesterol efflux [29]. Overexpression of ApoA-IV in the small intestine of transgenic mice has been reported to reduce aortic lesions and elevate high-density lipoprotein level [30]. The findings suggest that peripheral ApoA-IV plays an important role in the modulation of lipid metabolism. In the hypothalamus, ApoA-IV is colocalized with the POMC neurons in the arcuate nucleus (ARC) of the hypothalamus [20]. Central administration of ApoA-IV suppresses food intake through activation of the melanocortin system and inhibition of the neuropeptide Y (NPY) system [10,20,31]. When mice are maintained on standard chow diets, ApoA-IV global knockout (KO) mice have impaired lipid-induced NE synthesis and reduced UCP1-dependent BAT thermogenesis in response to dietary lipids [31]. After being fed a HFD for one week, ApoA-IV KO mice exhibit reduced diet-induced BAT thermogenesis and energy expenditure relative to their control groups [31]. These findings suggest that peripheral and/or central effects of ApoA-IV play an important role in the elevation of BAT thermogenesis and energy expenditure. Recently, we have reported that acutely intraperitoneal administration of ApoA-IV stimulates sympathetic activity and thermogenesis in BAT [28]. However, it is unclear whether central administration of ApoA-IV increases BAT thermogenesis. In the present studies, we tested the hypotheses that central administration of ApoA-IV stimulates BAT thermogenesis and that innervation of BAT is required for this action.

## 2. Results

### 2.1. Effect of Central ApoA-IV on BAT Thermogenesis

**Figure 1:**
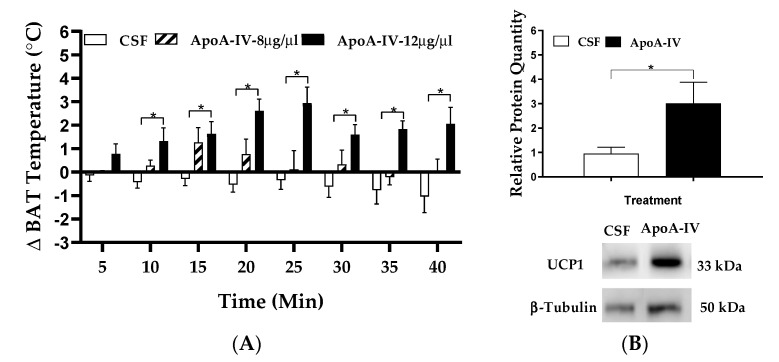
The effects of ApoA-IV on BAT temperature and UCP1 protein levels. BAT temperature was measured every 5 min for up to 40 min in mice following administration of either ApoA-IV at two different doses or CSF (**A**). The mice received central administration of either CSF (1 µL) or ApoA-IV (12 µg) into the third ventricle, and BAT was collected at 40 min post-injection. UCP1 protein in BAT was measured by immunoblot analysis (**B**). Data are expressed as the means ± SEM for 7–8 mice per group. * Represents a significant difference relative to the corresponding CSF-treated BAT (*p* < 0.5).

When animals received intracerebroventricular (ICV) administration of CSF, BAT temperature was lower than baseline starting at 10 min post-injection (*p* > 0.05, Figure 1A). Relative to CSF treatment, ICV injection of ApoA-IV protein at a dose of 12 µg, but not 8 µg, significantly increased BAT temperature starting at the 10 min post-injection timepoint (*p* < 0.05, Figure 1A). These observations suggested that ApoA-IV at 12 µg was an effective dose for induction of BAT temperature in mice.

Adenosine monophosphate-activated protein kinase (AMPK) is a major cellular energy sensor and its catalytic subunits, AMPKα1 and AMPKα2, are involved in modulation of thermogenesis in the adipose tissues [32,33]. Activation of the AMPK pathway leads to an increase in adipose triglyceride lipase (ATGL) and hormone-sensitive lipase (HSL), which are two key enzymes for intracellular TG hydrolysis in BAT. Additionally, this pathway leads to an increase in carnitine palmitoyltransferase 1 (CPT1) that facilitates fatty acid transfer into the mitochondria for fatty acid oxidation and induction of thermogenesis [34]. UCP1 and UCP3 expressed in BAT are involved in generation of heat production [35] and lipid metabolism [36,37]. To test if centrally administered ApoA-IV protein induces BAT thermogenesis, expression levels of thermogenic and lipolytic enzymes in BAT were determined. ApoA-IV at the dose of 12 µg significantly elevated UCP1 protein levels in comparison to CSF treatment (*p* < 0.05, Figure 1B).

**Figure 2:**
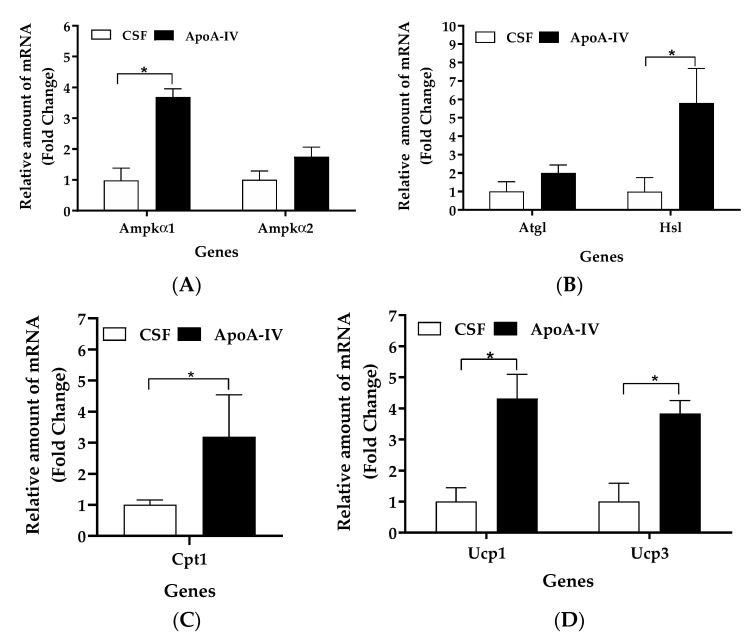
Lipolytic and thermogenic gene expression in adipose tissues. Levels of *Ampkα1* and *Ampkα2* (**A**), *Atgl* and *Hsl* (**B**), *Cpt1* (**C**), and *Ucp1* and *Ucp3* (**D**) gene expression were measured by qRT-PCR. Mice received central administration of either CSF (1 µL) or ApoA-IV (12 µg in 1 µL) into the third ventricle, and BAT was collected at 40 min post-injection. Data are expressed as the means ± SEM for 6–7 mice per group. * Represents a significant difference relative to CSF-treated controls (*p* < 0.05).

Compared to CSF treatments, ApoA-IV significantly increased gene expression levels of *Ampkα1*, *Hsl*, *Cpt1*, *Ucp1*, and *Ucp3* in BAT (*p* < 0.05, Figure 2A–D). No significant differences in *Ampkα2* and *Atgl* expression levels in BAT were observed between the CSF- and ApoA-IV-treated groups (Figure 2A,B).

Body weights and fat masses of BAT, epididymal white adipose tissue (EWAT), and inguinal white adipose tissue (IWAT) were similar between the CSF and ApoA-IV treatment groups (Table 1). Plasma levels of insulin, leptin, TG, and cholesterol in ApoA-IV-treated mice were comparable to those in CSF-treated mice (Table 1). These findings suggest that the elevated BAT thermogenesis induced by centrally administered ApoA-IV is independent of plasma insulin, leptin, and lipid levels.

### 2.2. Innervation of BAT Is Required for Central ApoA-IV-Induced Thermogenesis

**Figure 3:**
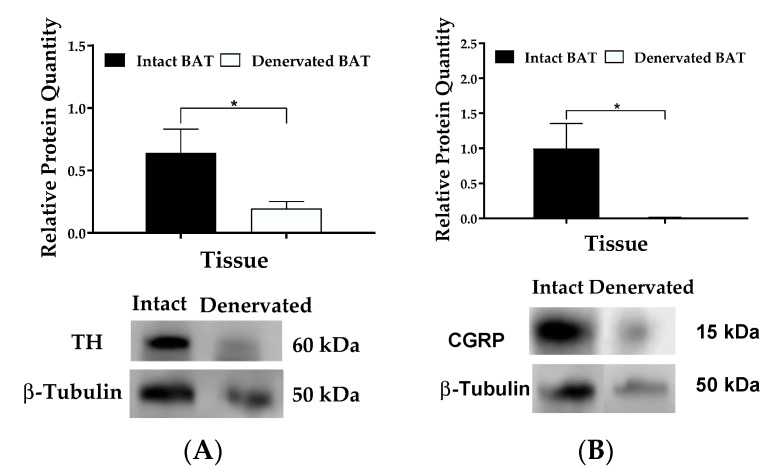
Levels of tyrosine hydroxylase (TH) and calcitonin gene-related peptide (CGRP) in innervated and denervated BAT. The TH (**A**) and CGRP (**B**) proteins were measured by immunoblot analyses. Mice with unilateral denervation of BAT received central administration of either CSF (1 µL) or ApoA-IV (12 µg in 1 µL) into the third ventricle, and BAT was collected at 40 min post-injection. Data are expressed as the means ± SEM for 10–11 mice per group. * Represents a significant difference relative to CSF-treated BAT (*p* < 0.05).

Sympathetic and sensory nerves are present in BAT [38,39]. In peripheral sympathetic neurons, tyrosine hydroxylase (TH) is the rate-limiting enzyme for the synthesis of NE, and NE is released at nerve terminals when sympathetic neurons are stimulated [40]. Thus, the expression of the TH protein can be used as a marker for sympathetic innervation to verify successful denervation of sympathetic nerves [41,42]. Calcitonin gene-related peptide (CGRP), expressed in most primary sensory neurons, is a commonly used marker for sensory innervation [41]. In the present experiment, unilaterally denervated BAT had significantly lower levels of the TH and CGRP proteins than the contralateral intact BAT (*p* < 0.05, Figure 3), indicating that mice had successful denervation of sympathetic and sensory nerves in one side of BAT.

**Figure 4:**
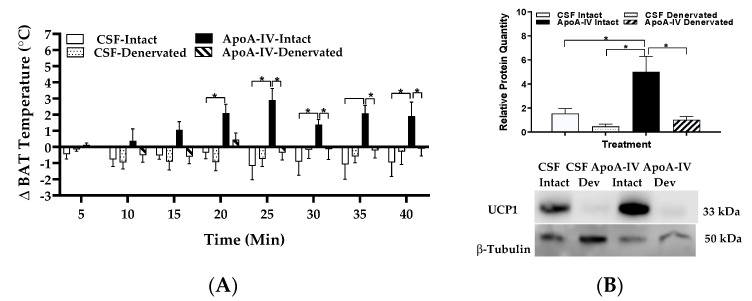
The effect of ApoA-IV on BAT temperature and UCP1 protein levels in mice with unilateral denervation of BAT. BAT temperature in intact and denervated BAT was measured every 5 min for up to 40 min in mice following administration of either ApoA-IV (n = 6–7/dose group) or CSF (**A**). Mice with unilateral denervation of BAT received central administration of either CSF (1 µL) or ApoA-IV (12 µg) into the third ventricle and BAT was collected at 40 min post-injection. UCP1 in BAT was measured by immunoblot analysis (**B**). Data are expressed as the means ± SEM for 5–6 mice per group. * Represents a significant difference relative to CSF-treated BAT (*p* < 0.05).

Relative to CSF treatment, ApoA-IV significantly elevated temperature in the intact BAT starting at 20 min after injection (*p* < 0.05, Figure 4A). In contrast, ApoA-IV-induced BAT temperature in the denervated BAT was lower than that in the intact BAT starting at 25 min after injection (*p* < 0.05, Figure 4A). In the intact BAT, ApoA-IV treatment significantly increased UCP1 protein expression relative to CSF treatment (*p* < 0.05, Figure 4B). In contrast, denervation of BAT significantly attenuated ApoA-IV-induced UCP1 protein (*p* < 0.05, Figure 4B). These findings are consistent with the findings in experiment 1 and suggest that surgical denervation diminishes ApoA-IV-induced UCP1-dependent BAT thermogenesis.

**Figure 5:**
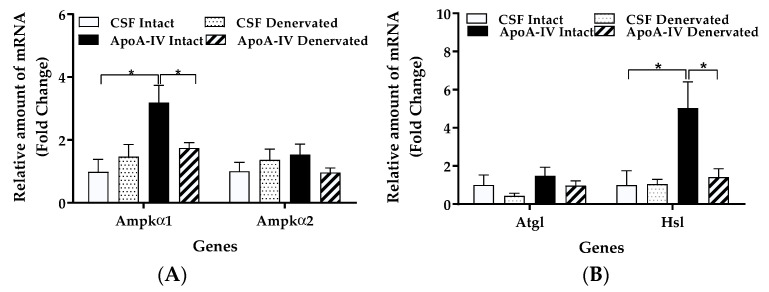

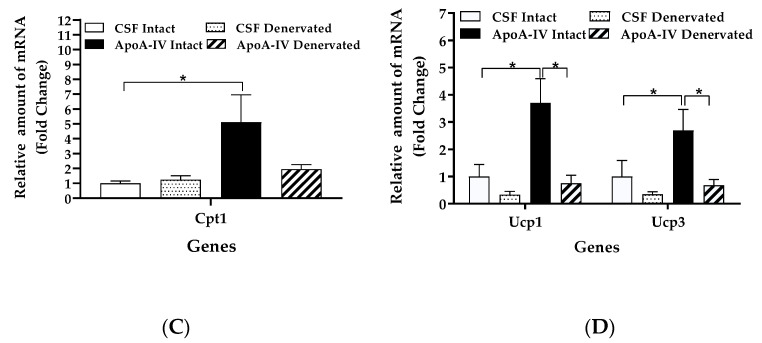
Lipolytic and thermogenic gene expression in intact and denervated BAT. Levels of *Ampkα1* and *Ampkα2* (**A**), *Atgl* and *Hsl* (**B**), *Cpt1* (**C**), and *Ucp1* and *Ucp3* (**D**) gene expression in intact and denervated BAT were measured by qRT-PCR. Mice with unilateral denervation of BAT received central administration of either CSF (1 µL) or ApoA-IV (12 µg) into the third ventricle, and BAT was collected at 40 min post-injection. Data are expressed as the means ± SEM for 6–8 mice per group. * Represents a significant difference relative to CSF-treated controls (*p* < 0.05).

Relative to CSF, ApoA-IV treatment enhanced gene expression levels of *Ampkα1*, *Hsl*, *Cpt1*, *Ucp1*, and *Ucp3* in intact BAT (*p* < 0.05, Figure 5A–D). In contrast, denervation reduced the ApoA-IV-induced increase in *Ampkα1*, *Hsl*, *Ucp1*, and *Ucp3*; the difference did not reach statistical significance for *Cpt1* (Figure 5A–D). Relative to CSF, ApoA-IV treatment did not increase *Ampkα2* or *Atgl* mRNA levels (Figure 5A,B). These findings suggest that central administration of ApoA-IV into the third ventricle elevates UCP1-dependent thermogenesis in BAT, and innervation is required for this action.

Body weights and fat masses of BAT, EWAT, and IWAT in mice with unilateral denervation of BAT were similar between the CSF and ApoA-IV treatments (Table 2). Similar to the mice that did not receive any surgery in experiment 1, ApoA-IV-treated mice with unilateral BAT denervation had comparable plasma levels of insulin, leptin, TG, cholesterol, and non-esterified Fatty acids (NEFA) to CSF-treated counterparts in experiment 2 (Table 2). These findings suggest that ApoA-IV treatment does not alter body weight, fat mass, or plasma parameters.

## 3. Discussion

Short-term consumption of dietary lipids increases ApoA-IV levels, activates β3-Adrenergic receptor signaling to increase intracellular lipolysis, and activates UCP1-dependent heat production in the mitochondria of BAT [22,43]. BAT contains numerous mitochondria and dense sympathetic innervation, and it is highly specialized for stimulated energy expenditure; thus, it plays an important role in the regulation of energy balance [43]. Both UCP1 and UCP3, which are involved in the regulation of adaptive thermogenesis [44,45,46,47], are expressed in BAT [48]. UCP1 has a half-life of 30 h while the half-life of UCP3 is 30 min [49]. The level of UCP1 is 400-fold higher than that of UCP3 in BAT [50]. Thus, UCP1 plays a key role in BAT thermogenesis. In the present experiment, central administration of ApoA-IV into the third ventricle of mice elevated BAT temperature and increased UCP1 and UCP3 expression in the BAT with intact innervation (in experiment 1 and sham-operated BAT in experiment 2), suggesting that ApoA-IV acts in the brain to increase BAT thermogenesis. UCP1 and UCP3 are mediators of thermogenesis regulated by β3-adrenergic stimulation [51,52]. Beta-adrenergic stimulation activates the AMPK signaling pathway, which leads to the activation of lipolytic enzymes for intracellular lipolysis and the generation of heat production [15,32,53,54] AMPK is a fuel-sensing enzyme complex that contains two catalytic subunits, α1 and α2, and is activated by increased adenosine monophosphate (AMP) or depleted adenosine triphosphate (ATP) [55,56]. Activation of AMPK increases adipose lipolysis through phosphorylation of ATGL and HSL [57].

ATGL is highly specific for TG hydrolysis whereas HSL hydrolyzes a much broader substrate spectrum, including diacylglycerols, cholesteryl esters, and retinyl esters [58,59] along with TG in lipid droplets. It is noteworthy that phosphorylated HSL is the activated form of HSL. Phosphorylated HSL protein level, however, is hardly detectable in BAT of lean rodents fed with a standard chow diet [26,59], possibly due to minimal accumulation of the substrates in BAT of lean animals. ATGL can be phosphorylated but, in contrast to HSL, the modification of ATGL phosphorylation may not lead to stimulated lipolysis [36]. Gene expression of *Atgl* is a sensitive way to indicate the ATGL protein level in cells. For example, the mRNA level of *Atgl* is first detected four days after induction of differentiation of murine 3T3-L1 adipocytes, and the maximum mRNA level of *Atgl* is observed six days after induction of differentiation [36]. Therefore, *Atgl* gene expression is a specific indicator for stimulated lipolysis in adipose tissue. The present study showed that ApoA-IV treatment increased gene expression levels of *Ampkα1* and *Hsl*, but not of *Ampkα2* or *Atgl*, in intact BAT, suggesting that although TG hydrolysis by ATGL is not enhanced, hydrolysis of cholesteryl and/or long-chain fatty acids esterified with retinyl esters by HSL may be increased. When the released fatty acids from intracellular lipolysis enter the mitochondria, CPT1 expressed in the outer mitochondrial membrane converts the fatty acyl group from CoA into carnitine [60] and promotes fatty acid oxidation [61] as well as BAT thermogenesis [32,62,63]. In intact BAT, ApoA-IV elevated *Cpt1* gene expression levels. These findings suggest that ApoA-IV-induced BAT thermogenesis is mediated through activation of an AMPKα1-dependent pathway for increased intracellular lipolysis and fatty acid oxidation.

Mild cold exposure is the most potent stimulus to activate BAT thermogenesis through stimulation of adrenergic receptor signaling pathways and BAT oxidative metabolism [64,65]. Recently, pharmacological activation of β2-adrenergic receptor signaling pathways has been reported to increase human BAT thermogenesis [66]. Mice have minimal unstimulated BAT activity at thermoneutrality [43]. The current experiments demonstrate that centrally administered ApoA-IV elevates BAT thermogenesis at 28–30 °C (thermoneutrality). In this regard, future studies to determine whether ApoA-IV stimulates human BAT thermogenesis at thermoneutrality as it occurs in mice will be crucial. Stimulation of BAT thermogenesis increases uptake of dietary fatty acids and glucose for replenishing energy substrates in BAT [67,68] and chronic induction of BAT thermogenesis by cold exposure results in elevation of food intake [69]. Thus, the major limitations to evoking BAT thermogenesis are compensatory upregulation in orexigenic pathways.

Surgical denervation produces nearly irreversible destruction of the sympathetic and sensory innervation of tissues [70]. Mice in the experiments reported here had unilaterally denervated BAT, i.e., one side of the intrascapular BAT was surgically denervated and its contralateral side was sham-operated as a within-animal control [71]. ApoA-IV-treated mice with the unilateral denervation of BAT had comparable body weight and fat mass to the CSF-treated group. In addition, ApoA-IV did not alter plasma TG, cholesterol, insulin, or leptin compared with CSF treatment. Thus, centrally administered ApoA-IV elevates UCP1-dependent BAT thermogenesis independent of effects of insulin and leptin. Consistent with previous observations [28], the present study showed that denervation attenuated ApoA-IV-induced BAT temperature and reduced levels of UCP, lipolytic enzymes, and AMPKα in the BAT. The findings suggest that sympathetic and sensory innervation is necessary for ApoA-IV-induced BAT thermogenesis.

Sympathetic activation increases the release of NE, the principal neurotransmitter released by sympathetic nerve terminals [72]. Subsequently, NE-induced β3-AR signaling stimulates lipoprotein lipase release from the capillary beds of adipose tissues into the circulation to hydrolyze TG [73]. Although direct effect of centrally administered ApoA-IV in the stimulation of sympathetic activity in BAT or white adipose tissues remains unknown, the present study shows that central administration of ApoA-IV does not alter plasma levels of TG, cholesterol, and NEFA, as well as of insulin and leptin in chow-fed mice. These findings suggest that central administration of ApoA-IV has a minimal effect on the regulation of lipid metabolism and incretin production in chow-fed mice. Further investigation of the effect of ApoA-IV in the regulation of lipid and glucose metabolism in obese mice is required.

ApoA-IV has been reported to increase the firing rate of POMC neurons, which is part of the central melanocortin system [10,74] that affects downstream sympathetic activity [75]. Thus, centrally administered ApoA-IV possibly acts on the melanocortin system to stimulate BAT thermogenesis. Further investigation of the involvement of the melanocortin system in ApoA-IV-induced BAT thermogenesis is required. Recently, we reported that intraperitoneal administration of ApoA-IV elevated sympathetic activity and enhanced BAT thermogenesis and that sympathetic and sensory innervation is necessary for the induction of BAT thermogenesis [28]. Our current findings, along with the findings of our previous study [31], suggest that both central and peripheral administration of ApoA-IV may stimulate UCP1-mediated BAT thermogenesis, and this action requires neural innervation network.

In the current study, BAT collected 40 min after ApoA-IV administration was investigated. Our results showed that *Ucp1* gene expression and UCP1 protein content were increased at this timepoint. It is possible that UCP1 mRNA and protein levels are persistently elevated for a longer period of time, which will be determined in future studies. Additionally, ApoA-IV administration may elevate BAT thermogenesis to a greater extent in obese animals with dampened sympathetic activity. Refeeding elevates the hypothalamic ApoA-IV level in fasted rodents when they are maintained on a chow diet or low-fat diet [22]. In contrast, HFD-induced obesity downregulates hypothalamic ApoA-IV, and refeeding fails to elevate hypothalamic ApoA-IV in fasted rodents fed a HFD [22]. Chronic ICV administration may elevate BAT thermogenesis and energy expenditure in obese animals, although time for persistent activation of BAT thermogenesis induced by central administration of ApoA-IV remains unknown in the current study. Further investigation for persistent elevation of BAT thermogenesis and energy expenditure induced by chronic administration of ApoA-IV and resulting in attenuation of body weight in obese mice is required.

## 4. Materials and Methods

### 4.1. Animals

Male C57BL/6J mice at 12 weeks of age were obtained from Jackson Laboratory (Bar Harbor, ME) and maintained in an Association for Assessment and Accreditation of laboratory Animal Care (AAALAC)-accredited facility at Ohio University on a 12 h–12 h light–dark cycle at 25 ± 0.5 °C. All animals were individually housed and maintained on a standard diet (14% fat, 8604, Envigo, Madison, WI, USA). Body weights and fat masses of BAT, EWAT, and IWAT were measured with a top-loading balance (Adenturer SL, Ohaus Corp, Pine Brook, NJ, USA). All animal protocols were approved by the Institutional Animal Care and Use Committees at Ohio University and the University of Cincinnati and followed the NIH Guide for the Care and Use of Laboratory Animals.

### 4.2. Mouse Recombinant ApoA-IV Protein

Mouse recombinant ApoA-IV protein was produced using a bacterial expression system as described in our previous report [74]. Briefly, ApoA-IV was expressed in a pET expression system and isolated from a sonicated cell extract using a nickel-chelating column. The His-tag on the ApoA-IV protein was removed using the IgA protease, and the mature protein was purified away from the cleaved tag by the second passage through the chelating column. The recombinant ApoA-IV protein has been shown to be of similar molecular mass as the ApoA-IV isolated from plasma. In addition, recombinant ApoA-IV protein was demonstrated to be as functional as the native form with regard to suppression of food intake and does not exert any adverse effect on food intake [76].

### 4.3. Animal Surgery

After acclimation and baseline measurements at 15 weeks of age, mice were anesthetized and received stereotaxic implantation of a cannula into the third ventricle. For the latter, anesthetized mice were placed in a stereotaxic apparatus and the height of the incisor bar was adjusted so that bregma and lambda had the same vertical coordinate. The tip of the vertically mounted 24-gauge stainless steel guide cannula (Plastic One Inc, Roanoke, VA, USA) was aimed at the third ventricle (coordinates were distance from bregma, −0.8 mm; distance from the midline, 0.2 mm; depth from dorsal surface, −4.8 mm).

After cannula implantation, the anesthetized mice received a midline skin incision on the back at the scapular region, and unilateral surgical denervation of the left or right side interscapular BAT was performed according to our established protocol [28,70]. Briefly, five intercostal nerves that exist in two major bundles were removed using fine-point microdissecting forceps on one side of BAT lobes. For sham surgeries, intact BAT (without denervation) contralateral to the denervated BAT lobe was gently moved with tissue forceps to visualize the nerves without damaging the nerves. Unilateral denervation allowed us to use the contralateral side of BAT as an internal control. Throughout denervation and sham surgeries, BAT pads were kept moist with saline-soaked gauze. After denervation, a temperature probe (BioMedic Data Systems, Inc., Seaford, DE, USA) was inserted beneath each of the right and left sides of intrascapular BAT [42] with a suture. The skin was closed with wound clips.

### 4.4. Experimental Designs

Mice have minimal unstimulated BAT activity at thermoneutrality (28–30 °C) [43]. On the experimental day, all mice were transferred to a procedure room at 28–30 °C prior to a 5 h fast. After the 5 h fast, mice received treatments in the procedure room at 28–30 °C prior to tissue collection.

In experiment 1, we tested whether ApoA-IV elevated BAT temperature in a dose-dependent manner. Seven days after probe implantation under the BAT, 5-h fasted mice without any denervation received an ICV injection via a cannula of either artificial CSF (1µL) or ApoA-IV protein (8 or 12 µg/µL in CSF, 1 µL) (n = 5 per group) into the third ventricle. To prevent BAT temperature change due to locomotor activity in free-moving animals, BAT temperature was recorded in anesthetized animals that were placed in the box with paper layers on the surface of heating pads according to a published protocol [77].

In experiment 2, we investigated the effects of ApoA-IV on BAT thermogenesis with and without innervation of BAT. Seven days after probe implantation under the BAT, a cohort of 5-h fasted mice (n = 7 per group) with unilateral BAT denervation received either CSF (1 µL) or ApoA-IV (12 µg/µL, 1 µL) into the third ventricle. BAT temperatures in the intact BAT and denervated BAT in the same animal were monitored using our IPTT-300 reader system (DAS-8007, BioMedic Data Systems) 15 min prior to treatments for baseline readings and every 5 min during the 40 min interval after an ICV injection of either ApoA-IV or CSF. The change in BAT temperature from baseline and at each timepoint was analyzed. At the end of the experiments, BAT and plasma were collected and stored at −80 °C for the measurements of lipid content, gene expression, and protein levels.

### 4.5. Determination of UCP1, TH, and Calcitonin Gene Related (CGR) Proteins

BAT proteins were extracted with a radioimmunoprecipitation assay (RIPA) lysis buffer system (Santa Cruz Biotechnology, Dallas, TX, USA), and total protein concentration was determined using the Bradford protein assay (Bio-Rad Laboratories, Hercules, CA, USA). For the measurement of UCP1 protein, extracted proteins (10 µg) were electrophoresed through a 4–20% acrylamide gel (Mini-PROTEAN precast protein gel, Bio-Rad Laboratories) with a tris/glycine/SDS running buffer at constant 100 V and then transferred to a polyvinylidene difluoride membrane (Bio-Rad Laboratories) for 1 h at a constant current of 350 mA. After blocking the membranes with a 5% blotting-grade blocker (non-fat dry milk, Bio-Rad Laboratories), membranes were then incubated at 4 °C overnight with primary rabbit or mouse polyclonal antibodies diluted 1:1000 in 5% bovine serum albumin (BSA) in tris-buffered saline: UCP1 (Abcam, Cambridge, MA, USA); TH and rabbit anti-CGRP (Cell Signaling Technology, Beverly, MA, USA); and mouse anti-β-tubulin (Invitrogen, Rockford, IL, USA) was included as an internal control [15,78]. After overnight incubation with the primary antibody, the immunoblots were washed and then incubated with the appropriate horseradish peroxidase-conjugated goat anti-rabbit antibody or rabbit anti-mouse antibody (1:5000 dilution, Dako Cytomation, CA, USA) for 1 h. Detection was achieved using an enhanced chemiluminescence system (Immobilon Western Chemiluminescent HRP Substrate, EMD Millipore Corporation, Billerica, MA, USA). A C-DiGit Blot Scanner (Li-Cor Biosciences, Lincoln, NE, USA) was used for visualization of the proteins, and quantification was performed using Image Studio Digit (LI-COR, version 5.2), normalizing all specific signals to β-tubulin.

### 4.6. Determination of BAT Gene Expression

Briefly, total BAT RNA was extracted using a PureLink RNA mini Kit (Thermo Fisher, Waltham, MA, USA) and cDNA was synthesized from 1 µg total RNA using an iScript cDNA synthesis kit (Hercules, CA, USA) [79]. Expression of UCP1 and UCP3, markers for the determination of heat production [80], and levels of CPT1, AMPKα1, and AMPKα2 were determined for fatty acid oxidation in BAT. Additionally, ATGL and HSL are markers for the determination of intracellular lipolysis in BAT. Levels of 36B4 mRNA for each sample were used as internal controls to normalize mRNA levels. The sequences of the primers (Integrated DNA Technologies, Coralville, IA, USA) are listed in Table 3. Quantitative real time-PCR (qPCR) was performed in a 25 µL final reaction volume with an Applied Biosystems StepOne Plus Real-Time-PCR instrument (Grand Island, NY, USA) using SYBR green RT-PCR master mixes (Life Technologies, Warrington, UK). Quantitative PCR conditions were as follows: 95 °C for 3 min for one cycle followed by 40 cycles of 95 °C for 30 s and 60 °C for 30 s. Threshold cycle readings for each of the unknown samples were used, and the results were analyzed in Excel using the ∆∆Ct method [22]. Expressions were normalized to *36B4* and presented relative to the CSF-treated intact BAT as 1.

### 4.7. Measurement of Plasma Parameters

Plasma TG, cholesterol, and NEFA levels in the plasma (5 µL) were determined using Infinity commercial assay kits (Thermo Scientific, Middletown, VA, USA) and a free fatty acid quantitation kit (Wako Diagnostics, Richmond, VA, USA), respectively, according to the manufacturers’ protocols. Absorbance was measured with a microplate reader (Synergy HT, BioTek Instruments, Inc, Richmond, VA, USA). Plasma insulin and leptin levels were determined using commercial ELISA kits (Millipore, St. Charles, MO, USA). Briefly, 10 µL plasma samples were added to each well of a microtiter plate pre-coated with anti-peptide monoclonal antibodies, and the detection antibody was added to the captured molecules. After incubation, absorbance was measured with a microplate reader (Synergy HT, BioTek Instruments), and the final concentrations were calculated using a series of dilutions of the standards provided with the ELISA kits.

### 4.8. Statistical Analysis

Significant differences between groups were determined by appropriate one-way analysis of variance (ANOVA) and two-way repeated measures ANOVA multiple comparison followed by Sidak test for multiple comparisons using GraphPad™ Prism (version 8.0, San Diego, CA, USA). All differences were considered to be significant if the *p*-values were <0.05.

## 5. Conclusions

Central administration of ApoA-IV into the third ventricle enhances BAT thermogenesis, and the sympathetic and sensory innervation is important for ApoA-IV-induced BAT thermogenesis. In addition, an AMPKα1-dependent pathway for elevated intracellular lipolysis and fatty acid oxidation appears to be involved in the induction of BAT thermogenesis. Furthermore, the action of ApoA-IV on BAT thermogenesis is independent of the effect of insulin and leptin.

## Figures and Tables

**Table 1:** Experiment 1: body weight, tissue weight, and levels of plasma parameters. After fasting for five hours, mice (n = 7/group) received intracerebral administration of either CSF (1 µL) or recombinant ApoA-IV protein at 12 µg/µL (1 µL). Plasma and tissues were collected 40 min after injections. Values are presented as the means ± SEM. BW: body weight; BAT: brown adipose tissue; EWAT: epididymal white adipose tissue; IWAT: inguinal white adipose tissue.

Treatments	CSF	ApoA-IV
BW (g)	26.4 ± 0.6	24.8 ± 0.6
BAT (g)	0.26 ± 0.17	0.15 ± 0.06
EWAT (g)	0.23 ± 0.04	0.29 ± 0.01
IWAT (g)	0.15 ± 0.04	0.15 ± 0.05
Insulin (ng/mL)	0.26 ± 0.02	0.28 ± 0.05
Leptin (ng/mL)	0.45 ± 0.08	0.62 ± 0.08
Triglyceride (ng/mL)	53.7 ± 10.20	51.03 ± 4.26
Cholesterol (ng/mL)	76.97 ± 8.80	80.22 ± 7.85

**Table 2:** Experiment 2: Body weight, tissue weights, and levels of plasma parameters. After fasting for five hours, mice (n = 7/group) received intracerebral administration of either CSF (1 µL) or recombinant ApoA-IV protein at 12 µg/µL (1 µL). Plasma and tissues were collected 40 min after injections. Values are presented as the means ± SEM. BW: body weight; BAT: brown adipose tissue; EWAT: epididymal white adipose tissue; IWAT: inguinal white adipose tissue.

Treatments	CSF	ApoA-IV
BW (g) prior to denervation	25.6 ± 1.0	26.9 ± 0.9
BW (g) after denervation	26.3 ± 0.3	27.4 ± 1.0
BAT (g)	0.07 ± 0.07	0.08 ± 0.01
EWAT (g)	0.13 ± 0.01	0.17 ± 0.01
IWAT (g)	0.16 ± 0.06	0.10 ± 0.01
Insulin (ng/mL)	0.24 ± 0.02	0.24 ± 0.03
Leptin (ng/mL)	0.59 ± 0.20	0.55 ± 0.14
Triglyceride (ng/mL)	46.00 ± 8.00	38.50 ± 6.70
Cholesterol (ng/mL)	71.00 ± 7.60	61.60 ± 12.10
NEFA (mmol/L)	0.46 ± 0.06	0.48 ± 0.05

**Table 3:** Primers for gene expression.

Primers	Forward 5′→3′	Reverse 5′→3′
Ucp1	ACTGGAGGTGTGGCAGTGTTC	ACGACCTCTGTAGGCTGCCCAA
Ucp3	GAGCGGACCACTCCAGCGTC	TGAGACTCCAGCAACTTCTC
Cpt1	ACCACTGGCCGAATGTCAAG	AGCGAGTAGCGCATGGTCAT
Ampkα1	CAGTAGGTACACACAGCGTAACACA	ACCTGTTACAGCAAATTCAAATGG
Ampkα2	TCCAGCACAGCTGAGAACCA	GGGATGCCGAGGACAAAGT
Atgl	GGTACCGTTCCCGAGGGAGACCAAGTGGA	CCTCGAGCGCAAGGCGGGAGGCCAGGT
Hsl	GCTTGGTTCAACTGGAGAGC	GGTAGAAGAGGGTCCATGAGG
36B4	ATCCCTGACGCACCGCCGTG	GCGCATCATGGTGTTCTTGC

## Abbreviations

HFD	High-fat diet
BAT	Brown adipose tissue
UCP1	Uncoupling protein 1
ApoA-IV	Apolipoprotein A-IV
POMC	Proopiomelanocortin
NE	Norepinephrine
NPY	Neuropeptide Y
ARC	Arcuate nucleus
KO	Knockout
TH	Tyrosine hydroxylase
CGRP	Calcitonin gene-related peptide
ICV	Intracerebroventricular
CPT1	Carnitine palmitoyltransferase I
AMPK	Adenosine monophosphate-activated protein kinase
ATGL	Adipose triglyceride lipase
HSL	Hormone-sensitive lipase
TG	Triglyceride
NEFA	Non-esterified FA
ANOVA	Appropriate one-way analysis of variance
qPCR	Quantitative real-time PCR
EWAT	Epididymal white adipose tissue
IWAT	Inguinal white adipose tissue
BSA	Bovine serum albumin
